# Polygenic Risk Score for Early Prediction of Sepsis Risk in the Polytrauma Screening Cohort

**DOI:** 10.3389/fgene.2020.545564

**Published:** 2020-11-12

**Authors:** Hongxiang Lu, Dalin Wen, Jianhui Sun, Juan Du, Liang Qiao, Huacai Zhang, Ling Zeng, Lianyang Zhang, Jianxin Jiang, Anqiang Zhang

**Affiliations:** ^1^State Key Laboratory of Trauma, Burns and Combined Injury, Wound Trauma Medical Center, Institute of Surgery Research, Daping Hospital, Army Medical University, Chongqing, China; ^2^Department of Traumatic Orthopaedics, General Hospital of Xinjiang Militarary Region, Urumuqi, China; ^3^College of Biomedical Engineering, Army Medical University, Chongqing, China

**Keywords:** trauma, sepsis, prediction, genetic variants, weighted genetic risk score

## Abstract

**Background:**

Increasing genetic variants associated with sepsis have been identified by candidate-gene and genome-wide association studies, but single variants conferred minimal alterations in risk prediction. Our aim is to evaluate whether a weighted genetic risk score (wGRS) that aggregates information from multiple variants could improve risk discrimination of traumatic sepsis.

**Methods:**

Sixty-four genetic variants potential relating to sepsis were genotyped in Chinese trauma cohort. Genetic variants with mean decrease accuracy (MDA) > 1.0 by random forest algorithms were selected to construct the multilocus wGRS. The area under the curve (AUC) and net reclassification improvement (NRI) were adopted to evaluate the discriminatory and reclassification ability of weighted genetic risk score (wGRS).

**Results:**

Seventeen variants were extracted to construct the wGRS in 883 trauma patients. The wGRS was significantly associated with sepsis after trauma (*OR* = 2.19, 95% CI = 1.53–3.15, *P* = 2.01 × 10^–5^) after being adjusted by age, sex, and ISS. Patients with higher wGRS have an increasing incidence of traumatic sepsis (*P*_trend_ = 6.81 × 10^–8^), higher SOFA (*P*_trend_ = 5.00 × 10^–3^), and APACHE II score (*P*_trend_ = 1.00 × 10^–3^). The AUC of the risk prediction model incorporating wGRS into the clinical variables was 0.768 (95% CI = 0.739–0.796), with an increase of 3.40% (*P* = 8.00 × 10^–4^) vs. clinical factor-only model. Furthermore, the NRI increased 25.18% (95% CI = 17.84–32.51%) (*P* = 6.00 × 10^–5^).

**Conclusion:**

Our finding indicated that genetic variants could enhance the predictive power of the risk model for sepsis and highlighted the application among trauma patients, suggesting that the sepsis risk assessment model will be a promising screening and prediction tool for the high-risk population.

## Introduction

Trauma is the fourth leading cause of death around the world. Despite advances in clinical management of trauma patients, major trauma results in approximately 15% of disabilities and 10% of deaths (Lord et al., 2014). Sepsis is one of the most serious complications post major trauma, which might result in progressive dysfunction of vital organs (Park et al., 2016). Sepsis and multiple-organ dysfunction syndromes (MODS) compound the systemic inflammation triggered by the original injury and are difficult to distinguish (Lindner et al., 2016). Therefore, early prediction of sepsis and then provision of the individual therapy accordingly are potential therapeutic managements of patients with traumatic sepsis (Eriksson et al., 2017).

Genetic variants of the immune system modulate the host response to infection (Hubacek et al., 2001; Eckert et al., 2013). Therefore, whether genetic heterogeneity might have significant impact on sepsis development is an important question. Evidences from animal experiments and human genetic association studies demonstrated that genetic heterogeneity contributed to a significant portion of susceptibility to sepsis (Wurfel, 2008; David et al., 2016). In recent years, increasing numbers of sepsis-predisposing variants have been identified by candidate gene and genome-wide association studies (GWAS) (Villar et al., 2004; Rautanen et al., 2015). For example, rs4957796 within the FER gene and rs5743551 located into the promoter region of TLR1 might affect the risk of sepsis (Thompson et al., 2014; Rautanen et al., 2015). Moreover, rs4919510 in MIR608 and rs2232618 in the coding region of the LBP gene were both functional variants and conferred susceptibility to sepsis after trauma (Zeng et al., 2012; Zhang et al., 2015). Therefore, delineating genetic heterogeneity for sepsis might contribute to the diagnostic approaches and therapeutic trials among trauma patients.

Due to the complex pathogenesis for development of sepsis, the prediction effect of the individual genetic variant was relatively limited. Previous studies suggested that great effect sizes might be obtained from genetic risk scores (GRS) comprising combined effects of multiple genetic variations (Jostins and Barrett, 2011; Chang et al., 2013; Abraham and Inouye, 2015). Therefore, we systematically reviewed all relevant studies and screened out the genetic variants potentially associated with risk of sepsis, and created the weight genetic risk score (wGRS) to evaluate the joint effect of multiple genetic variants for risk stratification of traumatic sepsis.

## Materials and Methods

### Patient Recruitment

In the present study, 1,000 major trauma patients were prospectively recruited. All patients were ethnic Han Chinese from the Department of Trauma Surgery in Daping Hospital between August 2010 and March 2016. Demographic and clinical information was derived from the electronic medical record and trauma biobank, as previously described (Zhang et al., 2015). Patients enrolled in the current study meet the following criteria: (1) aged from 18 to 65 years, (2) injury severity score (ISS) more than 16; and (3) survival ≥48 h in hospital. ISS was assessed based on the abbreviated injury score developed in 2005. As previously described, infection was considered to be a clinically obvious source or positive bacterial culture. Systemic inflammatory response syndrome (SIRS) was diagnosed based on two or more of the following conditions: body temperature <36°C or >38°C, heart rate > 90 beats per min, respiratory rate > 20 breaths per min, and white blood cell count <4,000 mm^3^ or >12,000 mm^3^ (Finkelsztein et al., 2017). The diagnosis criterion of sepsis was according to the Sepsis 2.0 for patients who met at least two of SIRS criteria plus infection (Jacome and Tatum, 2018). All patients were determined by two independent evaluators. Acute Physiology and Chronic Health Evaluation (APACHE) II and sequential organ failure assessment (SOFA) scores were carried out to evaluate the severity and organ failures after injury during the hospital days.

### Candidate Variant Selection

Eligible studies were searched from PubMed, Embase, Medline, Web of Science, and HuGE databases before March 13, 2016, by using the following keywords: associated or synonymous with “sepsis” and “polymorphism.” Moreover, we reviewed the full articles with the following criteria: (1) studies provided the number or frequency of genotypes in detail; and (2) these papers had an observational (case control or cohort) study design. The exclusion criteria were (1) studies with insufficient information; (2) abstract, comment, review, and editorial; and (3) for duplicate publications, only the most recent or complete study was included. Finally, 316 articles investigating 333 variants involving 147 distinct genes were included in the study (Supplementary Figure S1; Lu et al., 2019). Overall, we performed 334 meta-analyses on 65 variants with at least three study populations and identified 16 variants significantly associated with the risk of sepsis. Of the other 268 variants with fewer than three populations, 48 variants were identified according to the reported association results. Finally, 64 candidate variants were selected for further genotyping and analyzing (Supplementary Table S1).

### Genotyping and Quality Control

Blood specimens were obtained from trauma patients when admitted to the hospital within 24 h. The genomic DNA was extracted from whole blood using a genomic DNA purification kit (Promega, Madison, WI, United States). Genotyping was performed using the SNPscan method in all samples following the manufacturer’s instructions (Li et al., 2015). One blank control in each plate was used for genotyping quality control, and 10% of samples were duplicated. The overall concordance rate was 100% among the duplicated samples. The genetic variants with a calling rate of >96%, minor allele frequency (MAF) of >0.01, and Hardy–Weinberg equilibrium (HWE) at *P* > 0.01 in the overall trauma cohort were included for further analysis. To calculate the wGRS, only the patients that completely genotyped for all genetic variants were included for investigation.

### Statistical Analysis

Differences in categorical or continuous variables between sepsis and non-sepsis patients were compared using Pearson’s χ^2^-test or Student’s *t*-test, respectively. Deviation from the Hardy–Weinberg equilibrium (HWE) was tested using the χ^2^-test. The association between individual variant and sepsis was determined by logistic regression analyses. We defined a linear weight of 0, 1, and 2 to different genotypes containing 0, 1, and 2 risk alleles, respectively. Genetic variants with mean decrease accuracy (MDA) > 1 by the random forest algorithm were considered to have positive effects on the risk of sepsis and were chosen for construction of wGRS (Pisanu et al., 2017). The wGRS was constructed on the base of the β coefficients obtained from the logistic regression analysis in the additive model, and the equation was as follows: wGRS = β1 × SNP1 + β2 × SNP2 + … + βi × SNPi. The association between wGRS and sepsis risk was analyzed by unconditional logistic regression. Meanwhile, wGRS was divided into quartiles based on the distribution in the trauma cohort. Furthermore, the differences of SOFA and APACHE II score were calculated among different quartiles of wGRS. Finally, the joint effect of the significant clinical variables and wGRS was assessed to predict the sepsis after trauma through multivariate logistic regression model. The discriminative ability of wGRS was evaluated by receiver operating characteristic (ROC) curves and the areas under the curve (AUC). Net reclassification improvement (NRI) was applied to evaluate the ability of correct reclassification after adding wGRS to clinical variables.

The variance inflation factor (VIF) was performed to determine the collinearity of the multivariate logistic regression. Then, a nomogram was constructed according to the multivariate logistic regression model incorporating the selected variables. Decision curve analysis (DCA) was used to evaluate the clinical usefulness of the nomogram model by calculating the net benefits at different thresholds in the trauma cohort.

All statistical analyses were performed with SPSS 17.0 and R statistical software version 3.6.1. *P* < 0.05 was determined as statistical significance.

## Results

### Clinical Characteristics of Trauma Cohort

Demographic data of 1,000 trauma patients was summarized in Supplementary Table S2. Patients were mostly young (mean age: 42.89 ± 12.56 years) and severely injured (mean ISS: 19.59 ± 8.99). Incidence of sepsis was 26.20% (*n* = 262). Pneumonia and primary bloodstream infection was approximately 49.24% of all the documented infections. Gram-negative infections accounted for approximately 83.20%, gram-positive infections for 6.87%, and mixed gram-negative/gram-positive infections for 2.29% of sepsis patients. The median time for sepsis occurrence in the whole trauma cohort was 7.02 ± 6.95 days. The maximum of SOFA score and APACHE II score in hospital were 3.45 ± 2.79 and 8.27 ± 6.01, respectively. Twenty-one (2.10%) trauma patients died during the hospital days.

### Isolated Variants Have Only a Small Impact on Sepsis Risk

In the present study, 64 variants were successfully genotyped by the SNPscan method in 1,000 trauma patients. The overall calling rate was greater than 96%. All variants meet the criteria of MAF > 0.01 and *P*_(HWE)_ > 0.01 (Supplementary Table S3). Due to genotyping failure in some samples, 883 patients with complete genotyping data for all 64 variants were finally selected for further analysis. Firstly, we evaluated the association between 64 genetic variants and sepsis risk in additive genetic model using unadjusted logistic regression analysis (Supplementary Table S4). The results indicated that four variants were significantly related to the sepsis risk at a nominal level: rs2297518, located in the NOS2 gene (*OR* = 1.53, 95%CI = 1.12–2.10, *P* = 0.01); rs10865710, located in the PPARG gene (*OR* = 1.32, 95%CI = 1.06–1.63, *P* = 0.01); rs740598, located in the HSPA12A gene (*OR* = 1.25, 95%CI = 1.01–1.53, *P* = 0.04); and rs5743551, located in the TLR1 gene (*OR* = 1.26, *P* = 0.04). The associations of the four variants with sepsis were confirmed using logistic regression analysis, adjusting for age and sex. None of the other variants was associated with sepsis. These results indicted a relatively limited effect of single variants on sepsis in our trauma cohort.

### A wGRS Is Significantly Associated With Traumatic Sepsis

**FIGURE 1:**
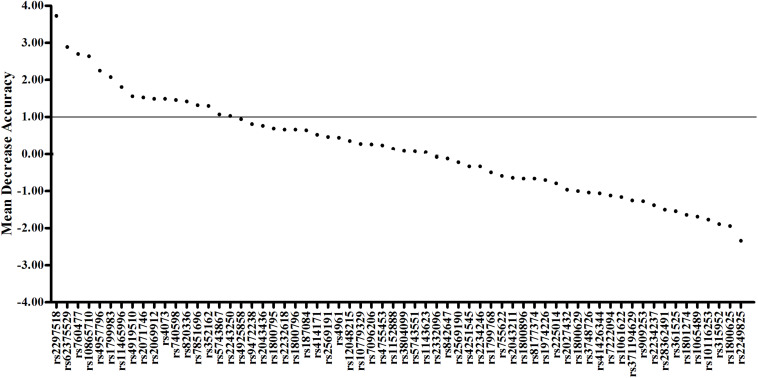
Random forest model including 17 variants previously associated with sepsis. The first 64 variables with the highest mean decrease accuracy are plotted. Seventeen sepsis-associated variants were shown to induce a positive change in mean decrease accuracy. These variants were thus considered to have a relevant influence on the model and were chosen for inclusion in the wGRS.

**FIGURE 2:**
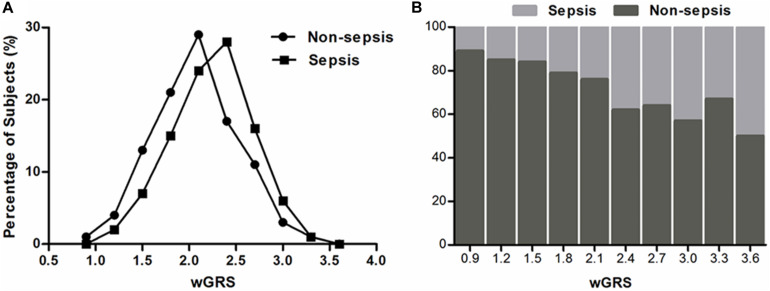
Distributions of the wGRS among sepsis cases and controls. **(A)** The percentage of wGRS of 17 variants displaying a significant difference among cases and controls. **(B)** The distributions of wGRS of 17 variants among cases and controls.

To evaluate the joint effect of these genetic variants on sepsis risk, a random forest algorithm was applied. As shown in Table 1 and Figure 1, 17 genetic variants induced a positive effect (MDA > 1) by random forest algorithm (Supplementary Table S4) were selected in the subsequent calculation of the wGRS. For all trauma patients, the wGRS distribution was ranging from 0.68 to 3.69. The incidence of sepsis increased significantly along with the increase of wGRS (Figure 2A), and cases had more risk alleles than controls (Figure 2B) using the wGRS of 17 variants (*P* = 3.47 × 10^–6^). As shown in Supplementary Table S5, unadjusted logistic regression analyses indicted the significant association between traumatic sepsis risk and wGRS (*OR* = 2.42, 95%CI = 1.73–3.39, *P* = 3.03 × 10^–7^), which was also significantly associated with sepsis after adjusted by age, sex, and ISS through multivariable logistic regression analysis (*OR* = 2.19, 95%CI = 1.53–3.15, *P* = 2.01 × 10^–5^).

**TABLE 1:** Seventeen selected genetic variants with MDA > 1 by random forest algorithm.

**Variant**	**Risk allele**	**Gene**	**MDA**	Beta*	**OR (95%CI)^#^**	***P***
rs2297518	G	NOS2	3.73	0.43	1.53 (1.12–2.10)	0.01
rs62375529	C	FER	2.89	0.29	1.34 (0.87–2.05)	0.19
rs760477	A	TONSL	2.70	0.03	1.03 (0.83–1.28)	0.97
rs10865710	G	PPARG	2.64	0.27	1.32 (1.06–1.63)	0.01
rs4957796	C	FER	2.25	0.29	1.34 (0.87–2.05)	0.19
rs1799983	T	NOS3	2.08	0.22	1.24 (0.93–1.70)	0.18
rs11465996	G	MD2	1.81	0.17	1.18 (0.92–1.52)	0.19
rs4919510	C	SEMA4G	1.56	0.05	1.05 (0.85–1.31)	0.63
rs2071746	T	HMOX1	1.53	0.07	1.07 (0.87–1.31)	0.53
rs2069912	C	PROC	1.49	0.06	1.07 (0.86–1.32)	0.56
rs4073	T	CXCL8	1.49	0.11	1.11 (0.90–1.38)	0.33
rs740598	G	HSPA12A	1.46	0.22	1.25 (1.01–1.53)	0.04
rs820336	G	MYLK	1.42	0.06	1.07 (0.62–1.85)	0.82
rs7851696	T	FCN2	1.32	0.14	1.15 (0.88–1.49)	0.31
rs352162	T	TLR9	1.30	0.12	1.13 (0.91–1.41)	0.27
rs5743867	C	TOLLIP	1.07	0.17	1.18 (0.95–1.46)	0.12
rs2243250	C	IL4	1.03	0.08	1.08 (0.83–1.40)	0.57

*^∗^The beta coefficient column shows natural logarithms of the ORs. ^#^Logistic regression in the additive model.*

We further classified all trauma patients (883 individuals) into four subgroups according to the wGRS quartiles: low-risk group (wGRS < 1.80), medium-risk group (wGRS = 1.80–2.20), high-risk group (wGRS = 2.20–2.50), and extremely high risk group (wGRS ≥ 2.50). The results demonstrated that 237 patients were classified into the low-risk group with 42 (17.72%) sepsis cases and 205 patients were classified into the extremely high-risk group with 83 (40.48%) sepsis cases. Compared with those individuals who had the lowest score (wGRS < 1.80), the trauma patients with a higher score had higher incidence of sepsis, with odds ratios of 1.47 (95%CI = 0.93–2.30, *P* = 0.10), 1.87 (95%CI = 1.20–2.92, *P* = 6.00 × 10^–3^), and 3.16 (95%CI = 2.05–4.88, *P* = 1.20 × 10^–7^), respectively (*P*_trend_ = 6.81 × 10^–8^) (Table 2). Furthermore, we compared the SOFA score and APACHEII score in different wGRS subgroups, which also demonstrated higher SOFA score (*P*_trend_ = 5.00 × 10^–3^) and APACHEII score (*P*_trend_ = 1.00 × 10^–3^) were observed in patients with higher score, respectively (Table 3).

**TABLE 2:** Cumulative effects of wGRS on the risk of sepsis.

**wGRS quartile**	**Patients, *n***	**ISS**	**Sepsis, *n* (%)**	**OR (95%CI)**	***P*_value_**	***P*_trend_**
(0.70–1.80) (≤ Q25)	237	20.28 ± 6.33	42 (17.72%)	1.00 (reference)		
(1.80–2.20) (Q25∼Q50)	225	21.26 ± 6.86	54 (24.00%)	1.47 (0.93–2.30)	0.10	
(2.20–2.50) (Q50∼Q75)	216	21.11 ± 7.57	62 (28.70%)	1.87 (1.20–2.92)	6.00 × 10^–3^	
(2.50–3.70) (> Q75)	205	21.22 ± 8.04	83 (40.48%)	3.16 (2.05–4.88)	1.20 × 10^–7^	6.81 × 10^–8^

*OR (95% CI), odds ratio (95% confidence interval).*

**TABLE 3:** Associations of wGRS with the severity and organ failure after trauma.

**wGRS quartile**	**Patients, *n***	**SOFA score^#^**	**APACHEII score^∗^**
(0.70–1.80) (≤ Q25)	237	3.05 ± 2.39	7.12 ± 5.23
(1.80–2.20) (Q25∼Q50)	225	3.46 ± 2.65	8.38 ± 6.40
(2.20–2.50) (Q50∼Q75)	216	3.62 ± 3.15	8.65 ± 6.61
(2.50–3.70) (> Q75)	205	3.98 ± 2.81	9.41 ± 5.86

*^#^P_trend_ = 5.00 × 10^–^^3^ among groups.*

*^∗^P_trend_ = 1.00 × 10^–^^3^ among groups.*

### Discriminative Ability for Traumatic Sepsis

**FIGURE 3:**
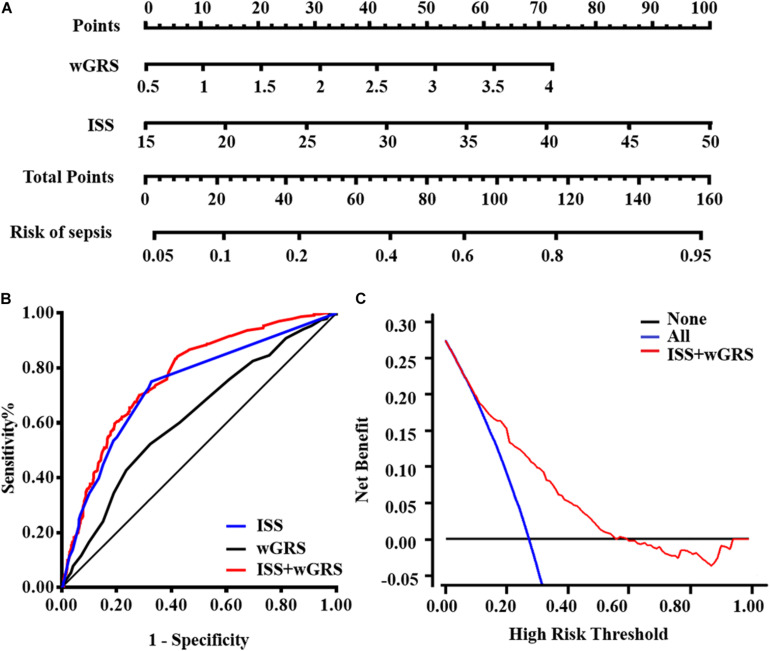
Model comparisons and clinical usefulness of the nomogram. **(A)** The nomogram incorporating ISS and wGRS was constructed for the prediction of sepsis after trauma. **(B)** ROC curves of three models for sepsis risk. The area under the ROC curves (AUCs) are based on logistic regression models of only clinical risk factor (ISS), only genetic factor (wGRS), and both clinical risk factor and genetic factor (ISS + wGRS). **(C)** DCA for the nomogram. The net benefit was plotted vs. the threshold probability. The red line represents the nomogram. The gray and black lines represent the hypothesis that all patients and no patients had sepsis, respectively.

wGRS and ISS were identified as independent risk predictors of sepsis in trauma patients using the multivariate logistic regression algorithm (Supplementary Table S5). Furthermore, the VIF of the two candidate predictors was 1.012, indicating that there was no collinearity. Therefore, wGRS and ISS were used to construct the prediction model of traumatic sepsis. To validate whether wGRS could enhance the predictive value, we conducted ROC to evaluate the predictive ability of three models: only ISS, only wGRS, and ISS plus wGRS. The AUCs of only wGRS and only ISS were 0.619 (95%CI = 0.586–0.651) and 0.734 (95%CI = 0.703–0.763), respectively. Our results demonstrated that when incorporating wGRS into the ISS, the AUC of the prediction model increased to 0.768 (95%CI = 0.739–0.796), with an increase of 3.40% (*P* = 8.00 × 10^–4^) (Figure 3B). To confirm the improvement, we considered NRI to estimate the reclassification of the prediction model when wGRS was included. Compared with the ISS, these reclassification rates gave an estimated NRI of 25.18% by including the wGRS into the ISS (95%CI = 17.84–32.51%, *P* = 6.00 × 10^–5^) (Table 4). Therefore, when wGRS was added to the clinical model, the ability of the prediction model improved significantly.

**TABLE 4:** Reclassification of predicted risk with the addition of wGRS using NRI.

**Predicted risk (without wGRS)**	**Reclassified risk (with wGRS)**					**N (%) of subjects reclassified**		**Net correctly reclassified**
	**<5%**	**5–10%**	**10–15%**	**15–20%**	**≥20%**	**Increased risk**	**Decreased risk**	
**Non-sepsis patients (*n* = 642)**								
0–5%	0	0	0	0	0	74 (11.53%)	217 (33.80%)	22.27%
5–10%	0	0	0	0	0			
10–15%	0	0	0	0	0			
15–20%	0	42	154	162	74			
≥20%	0	0	2	19	189			
**Sepsis patients (*n* = 241)**								
0–5%	0	0	0	0	0	26 (10.79%)	19 (7.88%)	2.91%
5–10%	0	0	0	0	0			
10–15%	0	0	0	0	0			
15–20%	0	1	11	22	26			
≥ 20%	0	0	3	4	174			
NRI(95%CI)						25.18% (17.84–32.51%)		
P						6.00 × 10^–5^		

### Clinical Usefulness of the Prediction Model

For the clinical usefulness, a nomogram incorporating the two predictors was constructed based on the multivariate logistic regression model that showed good calibration and discrimination in the trauma cohort (Figures 3A,B). The AUC of nomogram was 0.768, which was superior to either wGRS or ISS alone. As presented in Figure 3C, the DCA of the nomogram model indicated when the threshold probability is between 0 and 0.56, the nomogram performed more net benefit than the “treat-all” or “treat-none” strategies, which indicated that the nomogram was clinically useful.

## Discussion

In the current study involving 883 trauma patients, 17 of 64 potential risk variants identified systematically by previous GWAS and candidate gene association studies were used to calculate wGRS based on the random forest algorithm. Risk assessment models incorporating wGRS and ISS were a better tool to predict the risk value of traumatic sepsis. Our current study indicated that increased wGRS was significantly associated with a higher risk of traumatic sepsis. The model with only the ISS suggests low discriminatory accuracy (AUC = 0.734). However, when incorporating the wGRS based on 17 variants into the model, the AUC of the prediction model increases to 0.768 (*P* = 8.00 × 10^–4^), indicating that genetic predictors could enhance the prediction ability when added to the clinical factor model.

For major trauma patients, identifying those at high risk of sepsis then initiating appropriate treatment would improve the intensive therapy and clinical management (Brouwer et al., 2010; Lindner et al., 2016; Scherag et al., 2016; Taudien et al., 2016). Outcomes following major injury are affected by many factors, containing genetic variants, inflammatory response, immune dysfunction, coagulation dysfunction, tissue damage, and abnormal host responses to different pathogenic microorganisms. Recently, majority of detection scoring systems about sepsis focused on early and accurate sepsis diagnosis, such as *Insight* (Mao et al., 2018), SIRS, and SOFA, which were frequently changed during the outcome process of trauma. Many studies have indicated that genetic variants might be a major and stable factor for the prediction of sepsis risk. However, evidences also indicated that a single variant is not fully responsible for sepsis development (Namath and Patterson, 2011; Bronkhorst et al., 2015). In the study, we genotyped 64 genetic variants previously identified as susceptibility loci for sepsis risk. Multiple candidate genes of those polymorphisms were involved in pattern recognition receptors (PRRs), signal molecules, transcription factor, cytokines, and other immune regulated genes. PRRs are essential for recognition of microbial components and damage-associated molecular patterns and contribute to activation of the immune system (Kawai and Akira, 2010; Wiersinga et al., 2014). Therefore, those genetic variants exhibited a strong association with initiation and augmentation of sepsis (Arcaroli et al., 2005; Namath and Patterson, 2011), such as the TLR1-7202A/G (rs5743551) and TLR2 Asn248Ser (rs4833095) polymorphisms which have affected the function of TLR genes and TNFA-308G/A (rs1800629) and IL6-572C/G (rs1800796) which have affected the expression level of cytokine TNF-α and IL-6, respectively (Wurfel et al., 2008; Duan et al., 2011). Hence, genetic polymorphisms might be confirmed as potential beneficial biomarkers for evaluating sepsis risk in trauma patients. Furthermore, our data indicated these genetic biomarkers combined into the wGRS might improve the prediction accuracy.

To date, our study is the first attempt to construct and comprehensively evaluate the capacity of wGRS for risk prediction of traumatic sepsis. However, previous studies have indicated that genetic variants combined and/or into the traditional risk model could enhance the discriminatory capacity. For example, Jabandziev et al. (2014) demonstrated that specific combinations of five polymorphisms in the BPI (rs5743507), LBP (rs2232618), TLR4 (rs4986790), HSP70 (rs2227956), and IL-6 (rs1800795) genes appeared to predict the outcome of life-threatening sepsis in children. Shimada’s study (Shimada et al., 2011) indicated that the combined panel of TNFA-308G/A and IL1B-31C/T plus APACHE II score might enable a more accurate prediction of outcome in septic patients. Laurentiu et al. (David et al., 2016) summarized a few genetic variants observed in sepsis and suggested that specific genetic polymorphisms could be applied for early prediction of sepsis incidence in the future. In our previous study (Gu et al., 2010), we also indicated that eight functional polymorphisms (IL1B-1470, IL1B-511, IL1B-31, IL4-589, IL6-572, IL8-251, IL10-819, and TNFA-308) could be combined together to predict the risk of sepsis and organ dysfunction after trauma. In the current study, we revealed that the incidence of traumatic sepsis has been increased with the increase in wGRS. Genomic variants combined into wGRS could predict the risk of traumatic sepsis (AUC = 0.619), which was improved when adding the ISS factor (AUC = 0.768). To address the increasing discriminatory power, we studied the improved value of genetic factors to the clinical factor model by NRI (Pencina et al., 2011). The improvement in risk prediction of traumatic sepsis offered by wGRS was validated (Improved 25.18%) by a more detailed characterization and comparison between performances of models combined with genetic variants plus ISS factor together. DCA was recently considered as a novel analysis for evaluating clinical consequences the nomogram brings on decision-making. In our current study, DCA indicated that when thresholds are in the range between 0 and 0.56, decisions based on the nomogram would be applicable.

This study has several notable strengths. Firstly, our study established a risk prediction model through screening and evaluating genetic susceptibility from previous studies that have high prediction accuracy. Furthermore, genetic variants have several advantages as predictors, including remaining unchanged, predictable life-long risk, and easy, accurate, and cost-effective measurement (Jostins and Barrett, 2011; Muller et al., 2016). In addition, the combination of genetic and clinical factors into one model was feasible in clinical practice for trauma patients, which might enhance the discrimination of patients at high risk for sepsis. However, some limitations should be acknowledged. Firstly, in our current study, only ISS was significantly different between sepsis and non-sepsis trauma patients and included into the prediction model, but other risk factors (antibiotic usage, blood transfusion, and tracheal cannula et al.) could not be ignored in clinical practice (Lord et al., 2014), the prediction ability might be improved by adding these risk factors. Secondly, our sample size was relatively small and limited in the Chinese population. Whether our findings could be extended to the general or other ethnic population needed to be determined. Thirdly, we did not take into account possible gene–environment interactions or gene–gene interactions, but many interactions exist in reality.

## Conclusion

The current study investigates the risk predictive ability of accumulated genetic variants associated with traumatic sepsis in Chinese Han populations. The finding confirmed that trauma patients with a higher wGRS would be more susceptible to sepsis. When combined with other clinical factors, wGRS could improve the ability of personalized risk assessment for traumatic sepsis.

## Data Availability Statement

The datasets presented in this study can be found in online repositories. The names of the repository/repositories and accession number(s) can be found in the article/Supplementary Material. The datasets used and/or analyzed during the current study are available from the corresponding author on reasonable request.

## Ethics Statement

The studies involving human participants were reviewed and approved by the Ethical and Protocol Review Committees of Army Medical University. Written informed consent to participate in this study was provided by the participants’ legal guardian/next of kin.

## Author Contributions

JJ and AZ: conceptualization and supervision. HL, JS, DW, LQ, and HZ: methodology. HL, DW, and JD: formal analysis and investigation. HL and DW: writing – original draft preparation. HL, JJ, and AZ: writing – review and editing. JJ, LZe, and AZ: funding acquisition. LZe, LZh, and DW: resources. All authors contributed to the article and approved the submitted version.

## Conflict of Interest

The authors declare that the research was conducted in the absence of any commercial or financial relationships that could be construed as a potential conflict of interest.

## Supplementary Material

Supplementary Figure 1 The flow diagram for screening risk genetic variants from eligible studies.

## Abbreviations

ISS: injury severity score
MAF: minor allele frequency
HWE: Hardy–Weinberg equilibrium
MDA: mean decrease accuracy
wGRS: weight genetic risk score
SIRS: systemic inflammatory response syndrome
APACHE II: Acute Physiology and Chronic Health Evaluation II
SOFA: sequential organ failure assessment
MODS: multiple organ dysfunction syndrome
GWAS: genome wide association studies
AUC: area under the curve
ROC: receiver operating characteristic
AIC: Akaike’s information criterion
NRI: net reclassification improvement.
