# Supplementary figures and images for: Polygenic Risk Score for Early Prediction of Sepsis Risk in the Polytrauma Screening Cohort
Source: Front Genet. 2020 Nov 12;11:545564. doi: 10.3389/fgene.2020.545564 (PMC7689156; doi:10.3389/fgene.2020.545564)

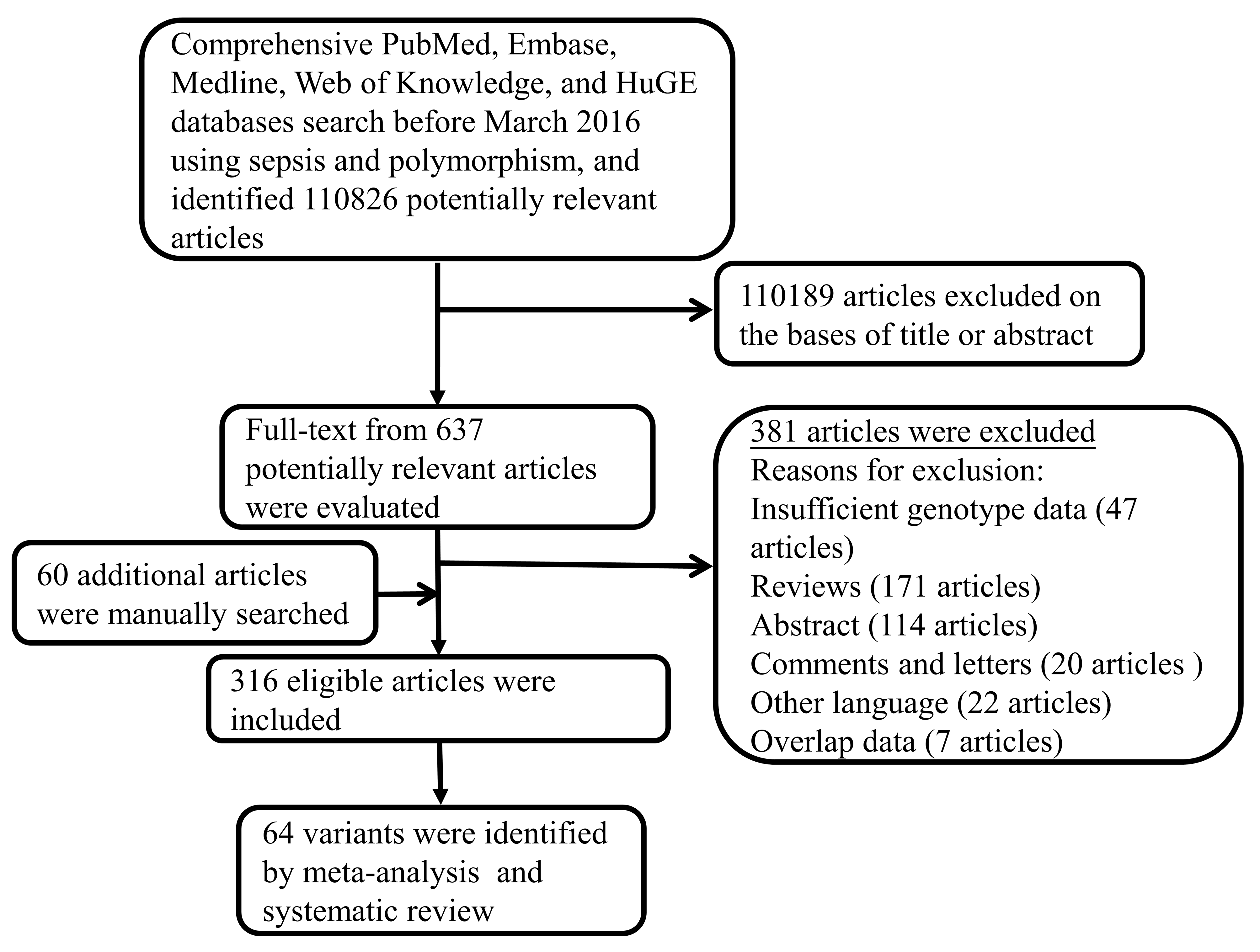

Supplement: Supplementary Figure 1 — The flow diagram for screening risk genetic variants from eligible studies. [file Image_1.TIF]
