# Supplementary material for: Polygenic Risk Score for Early Prediction of Sepsis Risk in the Polytrauma Screening Cohort
Source: Front Genet. 2020 Nov 12;11:545564. doi: 10.3389/fgene.2020.545564 (PMC7689156; doi:10.3389/fgene.2020.545564)
Supplement: Supplementary file 2 [file Data_Sheet_1.docx]

**Table S1. All selected 64 genetic variants associated with sepsis risk in previous literatures.**

| **Rs number** | **Chr** | **Position** | **Gene name** | **Region** | **Allele** | **CHBS_1000g** | **Classification** |
| --- | --- | --- | --- | --- | --- | --- | --- |
| rs1801274 | 1 | 161479745 | FCGR2A | exon | C/T | 0.32 | PRRs^*^ |
| rs1065489 | 1 | 196709774 | CFH | exon | G/T | 0.52 | Complement |
| rs1800896 | 1 | 206946897 | IL10 | 5'Flanking | A/G | 0.05 | Cytokines |
| rs2027432 | 1 | 247578441 | NLRP3 | 5'Flanking | C/T | 0.94 | PRRs |
| rs12048215 | 1 | 247584591 | NLRP3 | intron | A/G | 0.31 | PRRs |
| rs1061622 | 1 | 12252955 | TNFRSF1B | exon | G/T | 0.18 | Cytokines |
| rs10779329 | 1 | 218573741 | TGFB2 | intron | C/T | 0.69 | Others |
| rs842647 | 2 | 61119471 | REL | intron | A/G | 0.13 | TF^#^ |
| rs1143623 | 2 | 113595829 | IL1B | 5'Flanking | C/G | 0.40 | Cytokines |
| rs315952 | 2 | 113890304 | IL1RN | exon | C/T | 0.55 | Cytokines |
| rs2069912 | 2 | 128178191 | PROC | intron | C/T | 0.46 | TF |
| rs10865710 | 3 | 12353198 | PPARG | intron | C/G | 0.32 | TF |
| rs414171 | 3 | 50649499 | CISH | 5'UTR | A/T | 0.59 | Signal molecules |
| rs352162 | 3 | 52252969 | TLR9 | intron | C/T | 0.40 | PRRs |
| rs187084 | 3 | 52261031 | TLR9 | 5'Flanking | C/T | 0.40 | PRRs |
| rs2332096 | 3 | 121821143 | CD86 | intron | G/T | 0.28 | Others |
| rs820336 | 3 | 123415781 | MYLK | intron | A/G | 0.07 | Others |
| rs5743551 | 4 | 38807654 | TLR1 | 5'Flanking | A/G | 0.65 | PRRs |
| rs4073 | 4 | 74606024 | CXCL8 | 5'Flanking | A/T | 0.58 | Cytokines |
| rs3804099 | 4 | 154624656 | TLR2 | exon | C/T | 0.29 | PRRs |
| rs4961 | 4 | 2906707 | ADD1 | exon | G/T | 0.50 | Others |
| rs28362491 | 4 | 103422155 | NFKB1 | 5'Flanking | -/ATTG | 0.43 | TF |
| rs4957796 | 5 | 108402140 | FER | intron | C/T | 0.03 | Others |
| rs2569190 | 5 | 140012916 | CD14 | 5'UTR | A/G | 0.39 | PRRs |
| rs2569191 | 5 | 140013903 | CD14 | 5'Flanking | C/T | 0.39 | PRRs |
| rs62375529 | 5 | 108417332 | FER | intron | C/T | 0.03 | Others |
| rs2243250 | 5 | 132009154 | IL4 | 5'Flanking | C/T | 0.77 | Cytokines |
| rs909253 | 6 | 31540313 | LTA | intron | C/T | 0.48 | Cytokines |
| rs1800629 | 6 | 31543031 | TNF | 5'Flanking | A/G | 0.07 | Cytokines |
| rs361525 | 6 | 31543101 | TNF | 5'Flanking | A/G | 0.04 | Cytokines |
| rs1800625 | 6 | 32152442 | RAGE | 5'Flanking | C/T | 0.09 | PRRs |
| rs2234246 | 6 | 41243740 | TREM1 | 3'UTR | A/G | 0.22 | PRRs |
| rs2234237 | 6 | 41250466 | TREM1 | exon | A/T | 0.35 | PRRs |
| rs9472238 | 6 | 44214690 | HSP90AB1 | 5'Flanking | A/C | 0.37 | Others |
| rs1974226 | 6 | 52055335 | IL17A | 3'UTR | A/G | 0.06 | Cytokines |
| rs371194629 | 6 | 29798581 | HLA-G | 3'UTR | Del/Ins | 0.33 | Others |
| rs1800796 | 7 | 22766246 | IL6 | 5'Flanking | C/G | 0.75 | Cytokines |
| rs1800795 | 7 | 22766645 | IL6 | 5'Flanking | C/G | 1.00 | Cytokines |
| rs1799768 | 7 | 100769706 | PAI-1 | 5'Flanking | 5G/4G | 0.44 | Coagulation factors |
| rs1799983 | 7 | 150696111 | NOS3 | exon | G/T | 0.86 | Others |
| rs11465996 | 8 | 74901962 | MD2 | 5'Flanking | C/G | 0.23 | PRRs |
| rs760477 | 8 | 145668443 | TONSL | intron | G/A | 0.38 | TF |
| rs4925858 | 8 | 145671931 | NFKBIL2 | 5'Flanking | A/G | 0.39 | TF |
| rs10116253 | 9 | 120464320 | TLR4 | 5'Flanking | C/T | 0.40 | PRRs |
| rs41426344 | 9 | 120477933 | TLR4 | 3'UTR | C/G | 0.04 | PRRs |
| rs7851696 | 9 | 137779091 | FCN2 | exon | G/T | 0.18 | Others |
| rs4919510 | 10 | 102734778 | SEMA4G | intron | C/G | 0.58 | Others |
| rs7096206 | 10 | 54531685 | MBL2 | 5'Flanking | C/G | 0.85 | PRRs |
| rs740598 | 10 | 118506899 | HSPA12A | 5'Flanking | A/G | 0.50 | Others |
| rs5743867 | 11 | 1328351 | TOLLIP | intron | C/T | 0.62 | Signal molecules |
| rs8177374 | 11 | 126162843 | TIRAP | exon | C/T | 0.01 | Signal molecules |
| rs4755453 | 11 | 36530644 | TRAF6 | intron | C/G | 0.86 | Signal molecules |
| rs2043436 | 11 | 125925478 | CDON | intron | C/T | 0.30 | Others |
| rs4251545 | 12 | 44180295 | IRAK4 | exon | A/G | 0.11 | Signal molecules |
| rs1152888 | 12 | 66605228 | IRAK3 | exon | A/G | 0.61 | Signal molecules |
| rs2249825 | 13 | 31037903 | HMGB1 | intron | C/G | 0.17 | Cytokines |
| rs225014 | 14 | 80669580 | DIO2 | exon | C/T | 0.45 | Others |
| rs3748726 | 17 | 36557089 | SOCS7 | 3'UTR | A/G | 0.25 | Signal molecules |
| rs2297518 | 17 | 26096597 | NOS2 | exon | A/G | 0.15 | Others |
| rs7222094 | 17 | 43367653 | MAP3K14 | intron | C/T | 0.88 | Others |
| rs2043211 | 19 | 48737706 | CARD8 | exon | A/T | 0.48 | PRRs |
| rs2232618 | 20 | 37001761 | LBP | exon | C/T | 0.08 | PRRs |
| rs755622 | 22 | 24236392 | MIF | 5'Flanking | C/G | 0.21 | Cytokines |
| rs2071746 | 22 | 35776672 | HMOX1 | 5'Flanking | A/T | 0.53 | Coagulation factors |

*PRRs: Pattern recognition receptors; # TF: Transcription factors.

**Table S2. Characteristics of trauma cohorts.**

| **Demographics** | **Chongqing (n=1000)** |
| --- | --- |
| Age (yr) | 42.89±12.56 |
| Sex (male/female) | 812/188 |
| ISS | 19.59±8.99 |
| AIS max head/neck | 2.92±1.26 |
| AIS max face | 1.52±0.71 |
| AIS max thorax | 3.07±0.61 |
| AIS max abdomen | 2.64±0.98 |
| AIS max extremities/pelvis | 2.71±0.75 |
| GCS initial | 13.63±2.96 |
| GCS min | 13.51±3.14 |
| SOFA max | 3.45±2.79 |
| APACHE II max | 8.27±6.01 |
| Sepsis, n (%) | 262 (26.20) |
| Source of infection, n (%) |  |
| Respiratory tract infection | 70 (26.72) |
| Primary bloodstream infection | 59 (22.52) |
| Urinary tract infection | 63 (24.04) |
| Wound infection | 44 (16.79) |
| Other | 26 (9.92) |
| Gram-negative, n (%) | 218 (83.20) |
| Gram-positive, n (%) | 18 (6.87) |
| Mixed gram-negative and -positive, n (%) | 6 (2.29) |
| Others, n (%) | 18 (6.87) |
| Sepsis occurrence time (d) | 7.02±6.95 |
| Intensive care unit length of stay (d) | 13.73±7.04 |
| Hospital length of stay (d) | 33.29±30.19 |
| Initial (24h) pRBC transfusion (ml) | 1073.15±698.14 |
| Hospital mortality, n (%) | 21 (2.10%) |

**Table S3. Genotyping results among trauma patients.**

| **Variant** | **Call rate (%)** | **MA** | **MAF** | |  | **Genotypes, n** | | | **P_(HWE)_** |
| --- | --- | --- | --- | --- | --- | --- | --- | --- | --- |
|  |  |  | **CHBS_1000g** | **Trauma patients** |  | **Wild-type** | **Heterozygous** | **Variant** |  |
| rs10116253 | 98.80 | C | 0.40 | 0.39 |  | 369 | 472 | 147 | 0.99 |
| rs1061622 | 99.10 | G | 0.18 | 0.17 |  | 691 | 277 | 23 | 0.53 |
| rs1065489 | 99.10 | G | 0.48 | 0.48 |  | 213 | 515 | 263 | 0.23 |
| rs10779329 | 99.00 | T | 0.31 | 0.32 |  | 456 | 434 | 100 | 0.99 |
| rs10865710 | 98.90 | G | 0.32 | 0.35 |  | 428 | 436 | 125 | 0.41 |
| rs1143623 | 99.10 | G | 0.40 | 0.43 |  | 313 | 512 | 166 | 0.12 |
| rs11465996 | 99.10 | G | 0.23 | 0.20 |  | 639 | 305 | 47 | 0.15 |
| rs1152888 | 98.90 | A | 0.39 | 0.40 |  | 164 | 463 | 362 | 0.44 |
| rs12048215 | 99.10 | G | 0.31 | 0.33 |  | 448 | 429 | 114 | 0.46 |
| rs1799768 | 99.10 | 5G | 0.44 | 0.43 |  | 336 | 453 | 202 | 0.02 |
| rs1799983 | 99.10 | T | 0.14 | 0.11 |  | 782 | 193 | 16 | 0.39 |
| rs1800625 | 99.00 | C | 0.09 | 0.08 |  | 853 | 126 | 11 | 0.03 |
| rs1800629 | 99.10 | A | 0.08 | 0.07 |  | 871 | 112 | 8 | 0.09 |
| rs1800795 | 99.10 | C | 0.00 | 0.01 |  | 981 | 10 | 0 | 1.00 |
| rs1800796 | 99.10 | G | 0.25 | 0.26 |  | 56 | 408 | 527 | 0.04 |
| rs1800896 | 99.10 | G | 0.05 | 0.07 |  | 862 | 123 | 6 | 0.61 |
| rs1801274 | 99.10 | C | 0.32 | 0.33 |  | 449 | 434 | 108 | 0.80 |
| rs187084 | 99.10 | C | 0.40 | 0.35 |  | 427 | 439 | 125 | 0.51 |
| rs1974226 | 99.10 | A | 0.06 | 0.06 |  | 865 | 123 | 3 | 0.82 |
| rs2027432 | 99.10 | A | 0.06 | 0.07 |  | 4 | 129 | 858 | 0.64 |
| rs2043211 | 99.10 | T | 0.48 | 0.49 |  | 246 | 519 | 226 | 0.20 |
| rs2043436 | 99.10 | T | 0.30 | 0.29 |  | 485 | 438 | 68 | 0.02 |
| rs2069912 | 99.10 | C | 0.46 | 0.45 |  | 300 | 489 | 202 | 0.93 |
| rs2071746 | 99.10 | A | 0.47 | 0.46 |  | 215 | 476 | 300 | 0.35 |
| rs2232618 | 99.10 | C | 0.08 | 0.06 |  | 882 | 102 | 7 | 0.08 |
| rs2234237 | 99.10 | A | 0.35 | 0.30 |  | 476 | 432 | 83 | 0.33 |
| rs2234246 | 99.10 | A | 0.22 | 0.26 |  | 552 | 368 | 71 | 0.39 |
| rs2243250 | 99.10 | C | 0.23 | 0.21 |  | 39 | 344 | 608 | 0.37 |
| rs2249825 | 99.10 | C | 0.17 | 0.15 |  | 709 | 259 | 23 | 1.00 |
| rs225014 | 99.10 | C | 0.45 | 0.44 |  | 309 | 493 | 189 | 0.79 |
| rs2297518 | 99.10 | A | 0.15 | 0.15 |  | 711 | 254 | 26 | 0.62 |
| rs2332096 | 99.10 | G | 0.28 | 0.32 |  | 446 | 451 | 94 | 0.27 |
| rs2569190 | 99.00 | G | 0.39 | 0.40 |  | 363 | 472 | 155 | 0.98 |
| rs2569191 | 99.10 | T | 0.39 | 0.40 |  | 367 | 468 | 156 | 0.77 |
| rs28362491 | 96.30 | Del | 0.43 | 0.39 |  | 363 | 447 | 153 | 0.46 |
| rs315952 | 98.80 | T | 0.45 | 0.41 |  | 174 | 464 | 350 | 0.36 |
| rs352162 | 99.10 | C | 0.40 | 0.35 |  | 420 | 444 | 127 | 0.62 |
| rs361525 | 99.10 | A | 0.04 | 0.02 |  | 950 | 41 | 0 | 1.00 |
| rs371194629 | 98.70 | Ins | 0.33 | 0.34 |  | 426 | 445 | 116 | 1.00 |
| rs3748726 | 99.00 | G | 0.25 | 0.21 |  | 616 | 335 | 39 | 0.50 |
| rs3804099 | 99.00 | C | 0.29 | 0.29 |  | 508 | 389 | 93 | 0.13 |
| rs4073 | 99.10 | A | 0.42 | 0.44 |  | 189 | 504 | 298 | 0.40 |
| rs414171 | 99.10 | A | 0.41 | 0.39 |  | 178 | 469 | 374 | 0.93 |
| rs41426344 | 99.10 | C | 0.04 | 0.04 |  | 920 | 68 | 3 | 0.31 |
| rs4251545 | 99.10 | A | 0.11 | 0.10 |  | 803 | 169 | 19 | 0.01 |
| rs4755453 | 99.00 | C | 0.14 | 0.14 |  | 14 | 243 | 733 | 0.29 |
| rs4919510 | 99.10 | C | 0.42 | 0.46 |  | 204 | 511 | 276 | 0.36 |
| rs4925858 | 99.10 | A | 0.39 | 0.39 |  | 362 | 477 | 152 | 0.95 |
| rs4957796 | 99.10 | C | 0.03 | 0.06 |  | 872 | 118 | 1 | 0.22 |
| rs4961 | 99.00 | T | 0.50 | 0.49 |  | 269 | 481 | 240 | 0.40 |
| rs5743551 | 99.10 | T | 0.35 | 0.38 |  | 137 | 483 | 371 | 0.38 |
| rs5743867 | 99.00 | G | 0.38 | 0.39 |  | 156 | 460 | 374 | 0.46 |
| rs62375529 | 99.10 | C | 0.03 | 0.06 |  | 872 | 118 | 1 | 0.22 |
| rs7096206 | 99.10 | G | 0.15 | 0.17 |  | 31 | 276 | 684 | 0.71 |
| rs7222094 | 99.10 | T | 0.12 | 0.11 |  | 14 | 192 | 785 | 0.61 |
| rs740598 | 99.10 | G | 0.49 | 0.44 |  | 204 | 473 | 314 | 0.24 |
| rs755622 | 96.30 | C | 0.21 | 0.21 |  | 594 | 330 | 39 | 0.56 |
| rs760477 | 96.90 | A | 0.38 | 0.38 |  | 376 | 443 | 150 | 0.33 |
| rs7851696 | 99.00 | T | 0.18 | 0.17 |  | 680 | 275 | 35 | 0.31 |
| rs8177374 | 97.10 | T | 0.01 | 0.01 |  | 950 | 20 | 1 | 0.23 |
| rs820336 | 98.80 | A | 0.07 | 0.04 |  | 910 | 77 | 1 | 1.00 |
| rs842647 | 99.10 | A | 0.13 | 0.13 |  | 761 | 210 | 20 | 0.27 |
| rs909253 | 99.00 | C | 0.48 | 0.47 |  | 287 | 476 | 227 | 0.27 |
| rs9472238 | 99.10 | A | 0.37 | 0.37 |  | 407 | 443 | 141 | 0.26 |

**Table S4. Associations of single variant with traumatic sepsis by random forest algorithm and logistic regression analysis.**

| **Variants** | **Risk allele** | **MDA** | **Beta*** | **OR* (95%CI)** | **P** | **Adj OR^#^ (95%CI)** | **Adj P^#^** |
| --- | --- | --- | --- | --- | --- | --- | --- |
| rs2297518 | G | 3.73 | 0.43 | 1.53 (1.12-2.10) | 0.01 | 1.54 (1.13-2.10) | 0.01 |
| rs62375529 | C | 2.89 | 0.29 | 1.34 (0.87-2.05) | 0.19 | 1.35 (0.88-2.08) | 0.17 |
| rs760477 | A | 2.70 | 0.03 | 1.03 (0.83-1.28) | 0.97 | 1.04 (0.84-1.29) | 0.73 |
| rs10865710 | G | 2.64 | 0.27 | 1.32 (1.06-1.63) | 0.01 | 1.31 (1.05-1.62) | 0.02 |
| rs4957796 | C | 2.25 | 0.29 | 1.34 (0.87-2.05) | 0.19 | 1.35 (0.88-2.08) | 0.17 |
| rs1799983 | T | 2.08 | 0.22 | 1.24 (0.93-1.70) | 0.18 | 1.25 (0.91-1.72) | 0.17 |
| rs11465996 | G | 1.81 | 0.17 | 1.18 (0.92-1.52) | 0.19 | 1.18 (0.92-1.52) | 0.19 |
| rs4919510 | C | 1.56 | 0.05 | 1.05 (0.85-1.31) | 0.63 | 1.06 (0.85-1.31) | 0.63 |
| rs2071746 | T | 1.53 | 0.07 | 1.07 (0.87-1.31) | 0.53 | 1.07 (0.87-1.31) | 0.55 |
| rs2069912 | C | 1.49 | 0.06 | 1.07 (0.86-1.32) | 0.56 | 1.07 (0.87-1.32) | 0.53 |
| rs4073 | T | 1.49 | 0.11 | 1.11 (0.90-1.38) | 0.33 | 1.11 (0.90-1.38) | 0.33 |
| rs740598 | G | 1.46 | 0.22 | 1.25 (1.01-1.53) | 0.04 | 1.25 (1.01-1.53) | 0.04 |
| rs820336 | G | 1.42 | 0.06 | 1.07 (0.62-1.85) | 0.82 | 1.06 (0.61-1.84) | 0.84 |
| rs7851696 | T | 1.32 | 0.14 | 1.15 (0.88-1.49) | 0.31 | 1.15 (0.89-1.50) | 0.29 |
| rs352162 | T | 1.30 | 0.12 | 1.13 (0.91-1.41) | 0.27 | 1.13 (0.90-1.40) | 0.30 |
| rs5743867 | C | 1.07 | 0.17 | 1.18 (0.95-1.46) | 0.12 | 1.18 (0.96-1.46) | 0.12 |
| rs2243250 | C | 1.03 | 0.08 | 1.08 (0.83-1.40) | 0.57 | 1.08 (0.83-1.40) | 0.57 |
| rs4925858 | G | 0.94 | 0.08 | 1.08 (0.87-1.34) | 0.49 | 1.09 (0.87-1.35) | 0.46 |
| rs9472238 | A | 0.81 | 0.11 | 1.12 (0.91-1.39) | 0.29 | 1.12 (0.91-1.39) | 0.29 |
| rs2043436 | T | 0.76 | 0.06 | 1.06 (0.84-1.35) | 0.63 | 1.07 (0.84-1.36) | 0.60 |
| rs1800795 | G | 0.69 | 0.28 | 1.32 (0.27-6.39) | 0.73 | 1.34 (0.28-6.49) | 0.72 |
| rs2232618 | C | 0.66 | 0.17 | 1.19 (0.79-1.79) | 0.41 | 1.19 (0.79-1.79) | 0.40 |
| rs1800796 | G | 0.66 | 0.02 | 1.02 (0.79-1.30) | 0.90 | 1.01 (0.79-1.29) | 0.96 |
| rs187084 | T | 0.64 | 0.14 | 1.15 (0.93-1.44) | 0.21 | 1.15 (0.92-1.43) | 0.22 |
| rs414171 | T | 0.52 | 0.15 | 1.16 (0.93-1.44) | 0.19 | 1.15 (0.93-1.43) | 0.20 |
| rs2569191 | C | 0.46 | 0.03 | 1.03 (0.84-1.28) | 0.76 | 1.04 (0.84-1.28) | 0.75 |
| rs4961 | G | 0.44 | 0.02 | 1.02 (0.83-1.26) | 0.83 | 1.03 (0.83-1.26) | 0.81 |
| rs12048215 | A | 0.35 | 0.07 | 1.08 (0.86-1.34) | 0.51 | 1.08 (0.86-1.34) | 0.52 |
| rs10779329 | C | 0.27 | 0.02 | 1.02 (0.82-1.28) | 0.83 | 1.03 (0.82-1.29) | 0.81 |
| rs7096206 | G | 0.26 | 0.17 | 1.19 (0.91-1.55) | 0.21 | 1.18 (0.90-1.54) | 0.23 |
| rs4755453 | C | 0.23 | 0.18 | 1.20 (0.88-1.62) | 0.24 | 1.19 (0.88-1.61) | 0.25 |
| rs1152888 | A | 0.14 | 0.01 | 1.01 (0.81-1.24) | 0.97 | 1.00 (0.81-1.24) | 0.98 |
| rs3804099 | T | 0.09 | 0.06 | 1.06 (0.85-1.33) | 0.63 | 1.05 (0.84-1.32) | 0.67 |
| rs5743551 | A | 0.08 | 0.23 | 1.26 (1.01-1.56) | 0.04 | 1.26 (1.01-1.57) | 0.04 |
| rs1143623 | C | 0.05 | 0.14 | 1.15 (0.92-1.43) | 0.22 | 1.15 (0.92-1.43) | 0.22 |
| rs2332096 | C | -0.08 | 0.23 | 1.26 (1.00-1.59) | 0.05 | 1.26 (1.00-1.59) | 0.06 |
| rs842647 | G | -0.12 | 0.11 | 1.12 (0.82-1.53) | 0.49 | 1.12 (0.82-1.54) | 0.48 |
| rs2569190 | A | -0.22 | 0.02 | 1.02 (0.83-1.27) | 0.82 | 1.03 (0.83-1.27) | 0.80 |
| rs4251545 | G | -0.33 | 0.15 | 1.17 (0.83-1.64) | 0.38 | 1.17 (0.83-1.64) | 0.37 |
| rs2234246 | G | -0.33 | 0.03 | 1.03 (0.81-1.31) | 0.79 | 1.03 (0.81-1.31) | 0.80 |
| rs1799768 | 4G | -0.49 | 0.16 | 1.18 (0.96-1.44) | 0.12 | 1.18 (0.96-1.45) | 0.12 |
| rs755622 | G | -0.59 | 0.14 | 1.15 (0.88-1.50) | 0.31 | 1.15 (0.88-1.50) | 0.31 |
| rs2043211 | A | -0.64 | 0.01 | 1.00 (0.81-1.25) | 0.98 | 1.00 (0.81-1.24) | 0.98 |
| rs1800896 | G | -0.66 | 0.07 | 1.07 (0.69-1.65) | 0.76 | 1.08 (0.70-1.68) | 0.72 |
| rs8177374 | C | -0.66 | 0.23 | 1.26 (0.48-3.32) | 0.64 | 1.28 (0.49-3.38) | 0.62 |
| rs1974226 | G | -0.70 | 0.18 | 1.20 (0.77-1.86) | 0.43 | 1.20 (0.77-1.87) | 0.18 |
| rs225014 | T | -0.79 | 0.12 | 1.13 (0.91-1.40) | 0.26 | 1.14 (0.92-1.41) | 0.24 |
| rs2027432 | T | -0.96 | 0.23 | 1.26 (0.85-1.87) | 0.25 | 1.27 (0.86-1.90) | 0.24 |
| rs1800629 | A | -1.00 | 0.09 | 1.10 (0.74-1.63) | 0.65 | 1.09 (0.73-1.61) | 0.69 |
| rs3748726 | A | -1.04 | 0.02 | 1.02 (0.78-1.33) | 0.90 | 1.01 (0.78-1.32) | 0.93 |
| rs41426344 | C | -1.06 | 0.09 | 1.09 (0.63-1.89) | 0.76 | 1.10 (0.63-1.90) | 0.75 |
| rs7222094 | C | -1.12 | 0.10 | 1.11 (0.79-1.55) | 0.56 | 1.12 (0.79-1.57) | 0.53 |
| rs1061622 | T | -1.16 | 0.03 | 1.03 (0.77-1.37) | 0.86 | 1.02 (0.77-1.36) | 0.90 |
| rs371194629 | Del | -1.25 | 0.04 | 1.04 (0.83-1.30) | 0.75 | 1.04 (0.83-1.32) | 0.73 |
| rs909253 | T | -1.27 | 0.07 | 1.08 (0.87-1.33) | 0.49 | 1.08 (0.87-1.33) | 0.49 |
| rs2234237 | A | -1.38 | 0.07 | 1.08 (0.85-1.36) | 0.54 | 1.08 (0.85-1.36) | 0.53 |
| rs28362491 | Ins | -1.50 | 0.11 | 1.11 (0.90-1.38) | 0.33 | 1.12 (0.90-1.38) | 0.31 |
| rs361525 | G | -1.54 | 0.38 | 1.47 (0.63-3.42) | 0.37 | 1.47 (0.63-3.42) | 0.37 |
| rs1801274 | C | -1.64 | 0.03 | 1.03 (0.82-1.29) | 0.80 | 1.03 (0.82-1.29) | 0.81 |
| rs1065489 | T | -1.69 | 0.13 | 1.14 (0.92-1.41) | 0.25 | 1.13 (0.92-1.41) | 0.25 |
| rs10116253 | C | -1.77 | 0.01 | 1.01 (0.81-1.26) | 0.96 | 1.01 (0.81-1.26) | 0.91 |
| rs315952 | T | -1.89 | 0.12 | 1.12 (0.91-1.38) | 0.28 | 1.12 (0.91-1.38) | 0.28 |
| rs1800625 | T | -1.94 | 0.01 | 1.01 (0.69-1.48) | 0.97 | 1.01 (0.68-1.49) | 0.97 |
| rs2249825 | C | -2.34 | 0.02 | 1.02 (0.76-1.37) | 0.90 | 1.02 (0.76-1.36) | 0.91 |

*The beta coefficients column shows natural logarithms of the ORs; ^#^Analyses adjusted for age and sex.

**Table S5. Risk factors for sepsis in trauma patients.**

| **Variables** | **Univariate logistic regression** | | **Multivariate logistic regression** | |
| --- | --- | --- | --- | --- |
|  | **OR (95% CI)** | **P** | **OR (95% CI)** | **P** |
| **wGRS** | 2.42 (1.73-3.39) | 3.03×10^-7^ | 2.19 (1.53-3.15) | 2.01×10^-5^ |
| **ISS** | 1.12 (1.09-1.14) | 3.56×10^-23^ | 1.11 (1.09-1.14) | 1.49×10^-21^ |
| **Age** | 1.19 (0.80-1.77) | 0.39 | 1.23 (0.80-1.89) | 0.36 |
| **Sex** | 1.00 (0.99-1.01) | 0.88 | 1.00 (0.99-1.02) | 0.92 |
